# Supplementary material for: Asymptomatic carriage of Plasmodium falciparum in children no longer targeted for seasonal malaria chemoprevention and with a history of exposure to this strategy: A cross sectional study in southern Senegal
Source: PLoS One. 2025 Mar 25;20(3):e0318037. doi: 10.1371/journal.pone.0318037 (PMC11936201; doi:10.1371/journal.pone.0318037)
Supplement: S1 Table — (DOCX) [file pone.0318037.s001.docx]

**S1 Table. This is the S1 Table Title.** Amplification program for the *P. f18S* gene specific for *Plasmodium falciparum*.

| **Steps** | **Temperature (°C)** | **TIME** | **Number of cycles** |
| --- | --- | --- | --- |
| Denaturation | 50 | 2 mn | 1 cycle |
| Denaturation | 95 | 20 s | 1 cycle |
| Hybridization | 95 | 1 s | 40 cycles |
| Elongation | 60 | 20 s |  |
