# Supplementary material for: Asymptomatic carriage of Plasmodium falciparum in children no longer targeted for seasonal malaria chemoprevention and with a history of exposure to this strategy: A cross sectional study in southern Senegal
Source: PLoS One. 2025 Mar 25;20(3):e0318037. doi: 10.1371/journal.pone.0318037 (PMC11936201; doi:10.1371/journal.pone.0318037)
Supplement: S1 Fig — (DOCX) [file pone.0318037.s002.docx]

**S1 Figure. This is the S1 Figure Title.** Flowchart of recruitment and enrolment of children in the study.
