# Supplementary material for: Asymptomatic carriage of Plasmodium falciparum in children no longer targeted for seasonal malaria chemoprevention and with a history of exposure to this strategy: A cross sectional study in southern Senegal
Source: PLoS One. 2025 Mar 25;20(3):e0318037. doi: 10.1371/journal.pone.0318037 (PMC11936201; doi:10.1371/journal.pone.0318037)
Supplement: S2 Table — (DOCX) [file pone.0318037.s003.docx]

**S2 Table. This is the S2 Table Title.** Distribution of enrolled children according to their socio-demographic characteristics and history of exposure to SMC between 2013 and 2016.

|  | Number | % (CI 95%) |
| --- | --- | --- |
| Region | | |
| - Kédougou | 65 | 28.8 (23.2-34.4) |
| - Kolda | 144 | 63.7 (59.7-67.7) |
| - Sédhiou | 17 | 7.5 (1.1-13.9) |
| Sex |  |  |
| - Male | 113 | 50 (43.5-56.5) |
| - Female | 108 | 47.8 (41.3-54.3) |
| - ND | 5 | 2.2 (0.3-4.1) |
| Age (years) |  |  |
| - 11 | 80 | 35.4 (29.2-41.6) |
| - 12 | 104 | 46 (39.5-52.5) |
| - 13 | 32 | 14.2 (9.6-18.8) |
| - 14 | 9 | 4 (1.4-6.6) |
| - ND | 1 | 0.4 (-0.4-1.2) |
| History of exposure to SMC |  |  |
| - 2013 | 76 | 33.6 (27.4-39.8) |
| - 2014 | 198 | 87.6 (83.3-91.9) |
| - 2015 | 87 | 38.5 (32.2-44.8) |
| - 2016 | 1 | 0.4 (-0.4-1.2) |
| N^o^. of years of exposure |  |  |
| - 2013 and 2014 | 52 | 23 (17.5-28.5) |
| - 2013, 2014 and 2015 | 19 | 8.4 (4.8-12) |
| - 2013, 2014, 2015 and 2016 | 1 | 0.4 (-0.4-1.2) |
