# Supplementary material for: Asymptomatic carriage of Plasmodium falciparum in children no longer targeted for seasonal malaria chemoprevention and with a history of exposure to this strategy: A cross sectional study in southern Senegal
Source: PLoS One. 2025 Mar 25;20(3):e0318037. doi: 10.1371/journal.pone.0318037 (PMC11936201; doi:10.1371/journal.pone.0318037)
Supplement: S3 Table — (DOCX) [file pone.0318037.s004.docx]

**S3 Table. This is the S3 Table Title.** Distribution of positive cases by sociodemographic characteristics and SMC exposure history.

|  | Positif (N=44) | % (IC 95%) |
| --- | --- | --- |
| Region | | |
| - Kédougou | 22 | 50 (42.5-57.5) |
| - Kolda | 21 | 47.7 (36.8-58.6) |
| - Sédhiou | 1 | 2.3 (-12.5-17.1) |
| Sex |  |  |
| - Male | 19 | 43.2 (28.6-57.8) |
| - Female | 24 | 54.5 (39.8-69.2) |
| - ND | 1 | 2.3 (-2.1-6.7) |
| Age (years) |  |  |
| - 11 | 15 | 34.1 (20.1-48.1) |
| - 12 | 21 | 47.7 (32.9-62.5) |
| - 13 | 6 | 13.6 (3.5-23.7) |
| - 14 | 1 | 2.3 (-2.1-6.7) |
| - ND | 1 | 2.3 (-2.1-6.7) |
| History of exposure to SMC |  |  |
| - 2013 | 21 | 47.7 (32.9-62.5) |
| - 2014 | 38 | 86.4 (81.2-91.6) |
| - 2015 | 16 | 36.4 (29.2-43.6) |
| - 2016 | 0 | 0 |
| N^o^. of years of exposure |  |  |
| - 2013 and 2014 | 15 | 34.1 (27-41.2) |
| - 2013, 2014 and 2015 | 6 | 13.6 (8.4-18.8) |
| - 2013, 2014, 2015 and 2016 | 0 | 0 |
